# DNA barcode reveals occurrence of threatened species and hidden diversity on Teleost fish trade in the Coastal Amazon

**DOI:** 10.1038/s41598-023-47063-2

**Published:** 2023-11-13

**Authors:** Paula Santana, Thais Martins, Ítalo Lutz, Josy Miranda, Raimundo da Silva, David Mesquita, Rita Martins, Ivana Veneza, Marcelo Vallinoto, Iracilda Sampaio, Grazielle Evangelista-Gomes

**Affiliations:** 1 Laboratório de Genética Aplicada (LAGA), Instituto de Estudos Costeiros (IECOS), Universidade Federal do Pará (UFPA), Campus Bragança, Alameda Leandro Ribeiro S/N, Aldeia, Bragança, PA CEP: 68.600-000 Brazil; 2 Universidade Federal do Oeste Pará (UFOPA), Campus Monte Alegre, Monte Alegre, Brazil; 3 Laboratório de Evolução (LEVO), Instituto de Estudos Costeiros (IECOS), Universidade Federal do Pará (UFPA), Campus Bragança, Bragança, Brazil

**Keywords:** Genetic techniques, Sequencing, Genetic markers, Sequencing, Genetics, Ichthyology

## Abstract

This study aimed to identify the teleost fish species sold in Bragança, a major fishing hub on the north coast of Brazil. The COI gene analysis was performed for the identification of fish species. The local market uses common names that are not accurate and do not reflect the diversity of the species. 204 sequences were obtained, with 119 haplotypes. 83 species were identified by comparing with public databases and constructing phylogenetic trees, with Carangidae being the most prevalent family. The study also found *Haemulon atlanticus*, *Menticirrhus cuiaranensis* and *Hoplias misioneira*, a newly described species from the Amazon basin, among the samples. Additionally, 73 commercial names were recorded, including 10 categories, and the illegal trade of *Epinephelus itajara* was detected. The DNA Barcode method proved to be effective for discriminating the species. The study highlights that common and commercial names are vague and underestimate the fish diversity, and that Brazil needs to revise its regulations for commercial and scientific names.

## Introduction

The North Brazilian coast spans the states of Amapá, Pará and Maranhão and is about 2500 km long^1^. This region is home to a rich diversity of fish, both marine and estuarine, that have high economic and social importance^2–5^. Various fishing fleets operate in this region and bring their catch to state of Pará^6^, where Bragança is one of the main fish markets^7^. Bragança is a strategic location for studying the coastal ecosystem, as it has a large area of mangroves, estuaries and rivers nearby, such as the Caeté River, which provides a constant flow of organic matter and nutrients to the marine environment,^8,9^, resulting in high productivity and a rich biodiversity in the region^8,9^. The latest survey conducted in the Caeté estuary recorded 120 fish species belonging to 48 families, of which 19 species were new records for the north coast of Brazil^10^.

The fishing activity is influenced by the great diversity of resources available. In the municipality, fish landings happen daily^6^ and the fish originate from two different fleets, the large-scale (industrial), which targets fish in the deeper areas of the continental shelf, and the small-scale (artisanal) fleet, which operates in the areas closer to the shore^9^. Both catch a high diversity of species that supply the regional, national, and international market^6,11^. A lot of fish species from different regions come to the Municipal market and the City Fair, where they are available for sale all year round. According to a recent survey of the fish trade in Bragança, there were 98 species sold under 103 different trade names^12^. However, the survey only used morphology to identify the species. The use of trade names makes it hard to know the true diversity of the fish being sold, because these names can change within and between regions^13^. Moreover, sometimes multiple species are sold under one name as a category^14^, or one species may have different trade names depending on the region^15^. This lack of precision can cause confusion about the identity of the fish being sold, as well as enable the trade of endangered species^16,17^ and even harm the consumer, since it may increase the chance of commercial fraud^18^.

The challenge of accurately identifying species is a barrier that hinders the assessment and monitoring of the status of exploited resources, because there is no consistency in the use of the common name by fishermen^19^ and, therefore, by traders and consumers. To address the difficulty of standardizing trade names, Normative Instruction No. 53 of September 1, 2020 (MAPA), established a correlation between common and scientific names for the main species of fish with commercial importance in Brazil. However, this list still has many generic terms and ambiguities^20^. In this way, the precise identification of commercialized fish is necessary, to know the real diversity of fish sold, a primary measure for the effective management of resources to promote sustainable fishing. Traditionally, the identification of species is performed based on morphological characters, however, the small number of specialists in various groups has made it difficult to register biodiversity^21^. In addition, morphological approaches began to present some limitations, such as identification errors due to phenotypic plasticity, cryptic species, and individuals in the early stages of life, which are generally not contemplated in identification keys^22^, problems are even more evident in groups where species are highly similar and/or in processed fish, where diagnostic characters are removed^18,23^.

In view of this, the use of alternative methodologies is essential, such as molecular tools that use DNA for species-specific identification, especially the initial portion of the mitochondrial gene Cytochrome Oxidase C—subunit I (COI), a tool widely used as DNA Barcoding^22^. Since its proposal, several works have been reported in the literature for fish identification, demonstrating its efficiency for species discrimination and understanding of the diversity offered in categories^14,16^.

Among Brazilian examples, we highlight^14^, who were able to identify seven different species of fish sold together under the name Acará, in the Amazon, and^16^, who identified fish from markets in southern Brazil and found, in addition to replacement cases, trade in endangered species. DNA Barcoding has been shown to be a powerful tool for identifying species and revealing the hidden diversity that may be overlooked by traditional methods^16,17,24^. In this study, we applied DNA Barcoding to investigate the diversity of Teleost fish sold in Bragança, a coastal city in northeastern Pará, Amazon region. We compared the common names used in the market with the actual species identified by DNA Barcoding. We also detected the presence of endangered and newly described Teleost fish species in the trade. Our findings provide valuable information for the conservation and management of the fishery resources, as well as for the consumers' awareness and education.

## Results

### Teleost diversity traded using DNA barcoding

We analyzed 500 base pairs of DNA from 204 fish samples and found 258 polymorphic sites. The final alignment did not contain any deletions, insertions or stop codons. We obtained 119 haplotypes and compared their sequences to public databases. Table 1 shows the haplotypes, their molecular identification based on genetic similarity, and the commercial names of the fish samples. The sequences are publicly available, codes OR459502-OR459617 and OR515260-OR515262.

**Table 1:** Genetic similarity results of the 119 haplotypes of the 82 fish species traded on the Amazon coast compared to sequences from public databases.

Haplotype	Sample	Commercial designation	Molecular identification		
			Identification NCBI/BOLD	Similarity (%) NCBI/BOLD	Access NCBI/BOLD
1 (3)	CAI02M54	“Caica” 01	*Mugil curema*	100%/100%	EU715465/ Private
			*Mugil rubrioculus*	99.8%/99.8%	JX185208/GBGCA5935
2 (1)	CAI06M137	“Caica” 02	*Mugil hospes*	99.8%/99.8%	JX185217/GBGCA5926
			*Mugil brevirostris*	99.79%/99.79%	MK388724/GBMNC15807
3 (1)	CAI04F116	“Caica” 02	*Mugil hospes*	100%/100%	JX185217GBGCA5926
			*Mugil brevirostris*	100%/100%	MK388724/GBMNC15807
4 (1)	CAI07M138	“Caica” 02	*Mugil hospes*	99.4%/99.4%	JX185217/GBGCA5926
			*Mugil brevirostris*	99.38%/99.38%	MK388724/GBMNC15807
5 (1)	CAI08M152	“Caica” 03	*Mugil trichodon*	99.6%/99.6%	GU225405/MEFM111
			*Mugil curema*	99.8%/99.8%	JQ060590/MFSP425
6 (1)	CAI01M34	“Caica”	*Mugil incilis*	99.8%/99.8%	JX185189/ANGBF7284
7 (1)	TAI01M135	“Tainha”	*Mugil incilis*	100%/100%	JX185189/ANGBF7284
8 (1)	TCH01M0136	“Tainha chata”	*Mugil curema*	99.6%/99.6%	GU225396/MEFM668
			*Mugil rubrioculus*	100%/100%	JX185208/GBGCA5931
9 (2)	CAM02F07	“Camorim”	*Centropomus parallelus*	100%/100%	JX124754/MFSP817
10 (1)	CAM11F301	“Camorim”	*Centropomus parallelus*	99.8%/99.8%	JX124754/MFSP817
11 (1)	CAM07M273	“Camorim”	*Centropomus udecimalis*	100%/100%	KJ641480/GBGCA9221
12 (1)	CAM09M275	“Camorim”	*Centropomus udecimalis*	99.8%/99.8%	KJ641480/GBGCA9221
13 (1)	CAM10M276	“Camorim”	*Centropomus ensiferus*	99.2% 99.80%	MW183491/private
14 (1)	CAM06A212	“Camorim”	*Centropomus ensiferus*	99.6%/100%	MW183491/private
15 (1)	CARX01F82	“Caraximbó”	*Carax crysos*	100%/100%	GU702375/ MFSP341
16 (1)	CARX03F131	“Caraximbó”	*Carax crysos*	99.6%/99.6%	GU702375/ MFSP341
17 (3)	GUAR01F277	“Guarajuba”	*Carax crysos*	99.8%/99.8%	GU702375/ MFSP341
18 (1)	CAR01F169	“Caraaçu”	*Lobote surinamensis*	99.4%/99.4%	MH883042/ ANGBF55050
19 (1)	PAR06M170	“Pargo”	*Lutjanus purpureus*	99.4%/99.4%	MK534323/ANGBF51140
20 (1)	PAR05M74	“Pargo”	*Lutjanus purpureus*	99.8%/99.8%	MK534323/ANGBF51140
21 (2)	DEN02F69	“Dentão”	*Lutjanus jocu*	100%/100%	KF633383/ANGBF38633
22 (1)	DEN03F156	“Dentão”	*Lutjanus jocu*	99.6%/99.6%	KF633383/ANGBF38633
23 (2)	CIO02F133	“Cioba”	*Lutjanus synagris*	100%/100%	KF633322/ANGBF38885
24 (2)	GUAI294	“Guaiuba”	*Ocyurus chrysurus*	100%/100%	KF633271/ANGBF38920
25 (2)	GUAI234	“Guaiuba”	*Ocyurus chrysurus*	99.6%/99.6%	KF633271/ANGBF38920
26 (1)	CAN01M132	“Canguiro”	*Trachinotus cayennensis*	Does not own /99.8%	does not own / private
27 (1)	CAN02M150	“Canguiro”	*Trachinotus falcatus*	100%/100%	JQ365600/MFSP396
28 (5)	PAM12F227	“Pampo”	*Trachinotus carolinus*	100%/100%	MK368587/GBMNB5784
29 (10)	PAM06F188	“Pampo”	*Hemicaranx amblyrhynchus*	100%/100%	JQ365383/MFSP430
30 (1)	PAM02F184	“Pampo”	*Hemicaranx amblyrhynchus*	99.4%/99.4%	JQ365383/MFSP430
31 (4)	PAM10F225	“Pampo”	*Trachinotus carolinus*	99.8%/99.8%	MK368587/GBMNB5784
32 (5)	PAM09F217	“Pampo”	*Peprilus crenulatus*	99.8%/99.8%	KU201549/ANGBF42159
33 (1)	PAM15F232	“Pampo”	*Trachinotus goodei*	100%/100%	GU702381/MFSP143
34 (5)	PAL03F294	“Palombeta”	*Chloroscombrus chysurus*	100%/100%	KY402305/ANGBF17305
35 (6)	BIR01F117	“Birrete”	*Hemicaranx amblyrhynchus*	99.4%/99.4%	JQ365383/MFSP430
36 (2)	PGA04F166	“Peixe galo”	*Selene vômer*	100%/100%	MK291371/GBMNB5642
37 (1)	PGA02F154	“Peixe galo”	*Selene setapinnis*	100%/100%	JQ365560/MFSP797
38 (1)	PGA03F155	“Peixe galo”	*Selene setapinnis*	99.8%/99.8%	JQ365560/MFSP797
39 (2)	TIM06F251	“Timbiro”	*Oligoplotes saurus*	99.8%/99.8%	GU225649/MXII171
40 (1)	XAR01M101	“Xareu”	*Carax hippos*	100%/100%	GU225561/MXII172
41 (1)	ARA01F179	“Arabaiana”	*Seriola rivoliana*	100%/100%	MN134657/FIGAL031
42 (3)	PAR01F142	“Paru”	*Chaetodipterus faber*	100%/100%	KT367909/ANGBF37588
43 (1)	BIQ02F02	“Biquara”	*Haemulon steindachneri*	99.8%/99.8%	KY402341/GBMNB5595
44 (1)	PPD01F61	“Peixe pedra doido”	*Anisotremus virginicus*	100%/100%	EU697524/GBGC7670
45(1)	PPD02F62	“Peixe pedra doido”	*Anisotremus virginicus*	99.8%/99.8%	EU697524/GBGC7670
46 (5)	PPE09F198	“Peixe pedra”	*Genyatremus luteus*	100%/100%	KY402336/ANGBF23475
47 (1)	NI02M171	“Não identificado”	*Haemulon steindachneri*	99.6%/99.6%	KY402341/GBMNB5595
48 (1)	NI01F245	“Não identificado”	*Haemulon parra*	99.58%/99.58	JQ741222/ANGBF3694
49 (1)	JIQ01F123	“Jiquiri”	*Conodon nobilis*	99.79%/99.79%	KY402351/ANGBF23461
50 (1)	JIQ03F191	“Jiquiri”	*Conodon nobilis*	99.79%/99.79%	KY402351/ANGBF23461
51 (2)	COR02M114	“Corvina”	*Cynoscion leiarchus*	100%/100%	KP331714/GBMIN94192
52 (2)	COR02F71	“Corvina”	*Cynoscion virescens*	99.4%/99.4%	HM424137/MFSP328
53 (2)	COR05F255	“Corvina”	*Cynoscion microlepidotus*	100%/100%	KP331713/GBMIN128668
54 (2)	NI03F214	“Não identificado”	*Menticirrhus sp*	99.8%/99.8%	MT154399/ does not own
55 (1)	PCU04F122	“Pescada cururuca”	*Micropogonias furnieri*	99.4%/99.4%	KY402395/ANGBF30434
56 (5)	PBR01F265	“Pescada branca”	*Cynoscion acoupa*	100%/100%	KP331710/GBMIN94191
57 (1)	PAMA01M51	“Pescada amarela”	*Cynoscion acoupa*	99.79%/99.79%	KP331710/GBMIN94191
58 (4)	SGR02F91	“Sete grudes”	*Nebris micros*	100%/100%	KP331693/GBMIN118821
59 (1)	PCA01F120	“Pau de cachorro”	*Menticirrhus americanos*	100%/100%	KY402393/ANGBF30416
60 (4)	PGO04F89	“Pescada gó”	*Macrodon ancylodon*	100%/100%	KP331678/ANGBF30412
61 (1)	SARD02F254	“Sarda”	*Opisthonema oglinum*	99.6%/99.6%	KY402282/ANGBF34634
62 (1)	SAR01F08	“Sardinha”	*Sardinella aurita*	99.8%/99.8%	MK871646/ does not own
63 (1)	SAR08F248	“Sardinha”	*Sardinella aurita*	99.6%/does not own	MK871646/ does not own
64 (1)	SAR06F126	“Sardinha”	*Cetengraulis edentulus*	100%/100%	MT407631/private
65 (1)	URI01F06	“Uritinga”	*Sciades proops*	Does not own /100%	does not own / private
66 (1)	URI02F27	“Uritinga”	*Sciades proops*	Does not own /99.80	does not own / private
67 (1)	URI06F128	“Uritinga”	*Sciades proops*	Does not own /100%	does not own / private
68 (1)	URIC03F143	“Uricica amarela”	*Cathorops spixii*	99.8%/99.8%	MF595235/private
69 (1)	URIC02F130	“Uricica amarela”	*Cathorops spixii*	100%/100%	MF595235/private
70 (1)	URIC06M129	“Uricica amarela”	*Cathorops spixii*	99.6%/99.6%	MF595235/private
71 (1)	URICB03F262	“Uricica branca”	*Sciades couma*	Does not own /99.80%	does not own / ITAPE389
			*Sciades herzbergii*	Does not own /99.80%	does not own /private
72 (1)	JUR01F279	“Jurupiraga”	*Amphiarius rugispinis*	Does not own /100%	does not have / ITAPE389
73 (1)	GUR04M237	“Gurijuba”	*Netuna/Sciades parkeri*	100%/100%	HQ689375 / private
74 (2)	BAN03M55	“Bandeirado”	*Bagre bagre*	100%/100%	GU702398/MFSP119
75 (5)	BAG02F139	“Bagre”	*Sciades couma*	Does not own /100%	does not own / ITAPE389
			*Sciades herzbergii*	Does not own 100%	does not own /private
76 (1)	BRAG02F290	“Bragalhão”	*Sciades couma*	Does not own /99.6%	does not own / ITAPE024
77 (1)	BRA01F289	“Bragalhão”	*Sciades couma*	Does not own /100%	does not own / ITAPE024
78 (1)	CANG01F146	“Cangatã”	*Notarius luniscutis*	99.6%/99.6%	JQ365226/MFSP344
79 (1)	CAMB01F180	“Cambeua”	*Sem correspondência*	Does not own /100%	does not own /private
80 (1)	BON01F92	“Bonito”	*Euthynnus alletteratus*	99.77%/99.77%	DQ835903/GBGC7883
81 (1)	CAV01F269	“Cavala”	*Scomberomorus cavala*	100%/100%	GU225658/MXII124'
82 (2)	SER04M286	“Serra”	*Scomberomorus brasiliensi*	100%/100%	JQ365547/MFSP844
83 (1)	SER02F268	“Serra”	*Scomberomorus brasiliensi*	99.6%/99.6%	JQ365547/MFSP844
84 (1)	ATU01M35	“Atum”	*Katsuwonus pelamis*	100%/100%	DQ835928/GBGC3285
85 (1)	ATU02M67	“Atum”	*Thunnus atlanticus*	100%/100%	GU225687/MXII119
86 (1)	ATU03M272	“Atum”	*Thunnus atlanticus*	99.8%/99.8%	GU225687/MXII119
87 (8)	GAR01F05	“Garoupa”	*Epinephelus Itajara*	100%/100%	KF836462/ANGBF40182
88 (1)	PIRAR01M113	“Pirarena”	*Cephalopholis fulva*	99.8%/99.8%	JQ365278/MFSP1897
89 (1)	PAC01F49	“Pacamum”	*no match*		does not own / does not own
90 (1)	PAC02F50	“Pacamum”	*no match*		does not own / does not own
91 (1)	BDO01F96	“Bico doce”	*Diapterus rhombeus*	99.8%/99.8%	KY402329/ANGBF38445
92 (1)	PVO01F175	“Peixe voador”	*Cheilopogon cyanopterus*	99.59%/99.59%	KU943241/ZOSKT383
93 (1)	PIRAP01F211	“Pirapema”	*Megalops atlanticus*	99.8%/99.8%	GU224551/MFL034-06
94 (1)	GUAR01F200	“Guaravilha”	*Trichiurus lepturus*	100%/100%	JX124915/MFSP806
95 (1)	TLH02F159	“Tralhoto”	*Anableps anableps*	99.03%/99.03%	LC154806/GBMIN132751
96 (1)	PES01F269	“Pescadinha”	*Plagioscion squamosissimus*	99.6%/99.6%	MZ052054/GBOL1623
97 (1)	DOU01M266	“Dourada”	*Brachyplatystoma rousseauxii*	100%/100%	FJ418759/ANGBF6854
98 (3)	TUV01F05	“Tuvi”	*Sternopygus macrurus*	99.6%/99.6%	MN195126/GBMNB9728
99 (1)	PAB01M14	“Paboca”	*Mylossoma duriventre*	99.6%/99.6%	MG752458/ANGBF33790
100 (1)	PAB02F102	“Paboca”	*Mylossoma duriventre*	99%/99%	MG752458/ANGBF33790
101 (1)	PACU02F222	“Pacu”	*Mylossoma duriventre*	99.8%/99.8%	MG752458/ANGBF33790
102 (1)	PIAB01F209	“Piaba”	*Astyanax bimaculatus*	Does not own /100%	does not own t/private
103 (1)	PIR01F21	“Piramutaba”	*Brachyplatystoma vaillantii*	100%/100%	MT551762/GBMND28106
104 (2)	PIR04F189	“Piramutaba”	*Brachyplatystoma vaillantii*	99.6%/99.6%	MT551762/GBMND28106
105 (4)	PIRA04F110	“Piranha”	*Pygocentrus nattereri*	100%/100%	MG752553/ANGBF33897
106 (1)	PIRA03F109	“Piranha”	*Pygocentrus nattereri*	99.6%/99.6%	MG752553/ANGBF33897
107 (1)	TIL01M93	“Tilapia”	*Oreochromis niloticus*	99.8%/99.8%	MK355381/ANGBF51171
108 (1)	TIL03M95	“Tilapia”	*Oreochromis niloticus*	100%/100%	MK355381/ANGBF51171
109 (1)	PIA04F86	“Piau”	*Schizodon fasciatus*	99.8%/99.8%	FJ440621/GBGCA131
110 (3)	ARA02F220	“Aracu”	*Schizodon fasciatus*	100%/100%	FJ440621/GBGCA131
111 (1)	TAMO01F15	“Tamoata”	*Hoplosternum littorale*	99.8%/99.8%	HM405079/BSB226
112 (1)	TAMO02F16	“Tamoata”	*Hoplosternum littorale*	99.6%/99.6%	HM405079/BSB226
113 (3)	TAM01F36	“Tambaqui”	*Colossoma macropomum*	100%/100%	HQ420846/ANGBF6843
114 (3)	MAN01F160	“Mandi”	*Pimelodus cf. argenteus*	98.8%/98.8%/	KP294272 / private
115 (1)	TRA01F75	“Traira”	*Hoplerythrinus unitaeniatus*	98%/98%	JN988902/private
116 (4)	TRA02F76	“Traira”	*Hoplias missioneira*	100%/100%	MG699541/private
117 (1)	BEJ01M296	“Bejupira”	*Rachycentron canadum*	100%/100%	EF609446/FOAC501
118 (2)	URR01A208	“Urrubaiana”	*Elops saurus*	100%/100%	GU702393/MFSP114
			*Elops smithi*	100%/100%	GU224783/MFLII064
119 (1)	ANC01M271	“Anchova”	*Pomatomus saltatrix*	100%/100%	JQ365515/MFSP793

In parentheses, the number of individuals sharing each haplotype.

**Figure 1:**
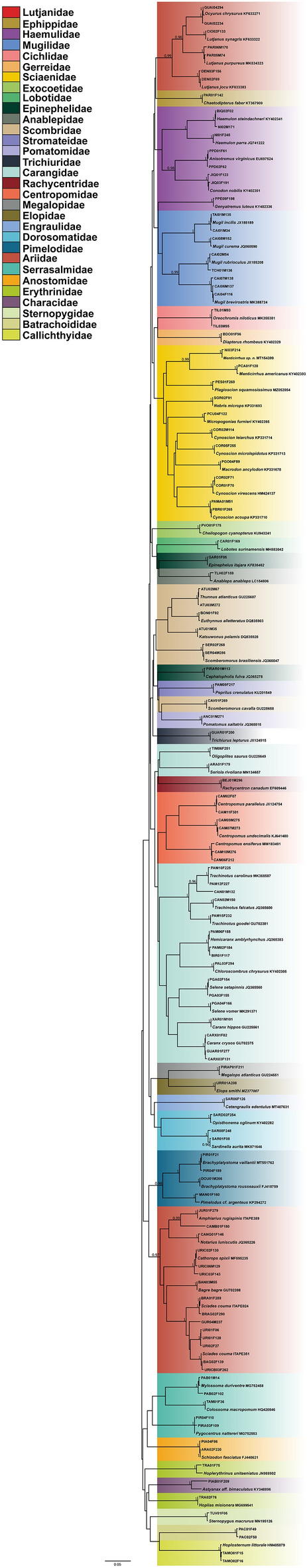
Neighbor joining tree (NJ) for the 119 haplotypes with reference bank, NCBI, and BOLD sequences. The numbers over the branches indicate Bootstrap's statistical support. The coloring of taxa was organized by family and '*' indicates threatened species.

We identified 82 species of teleost fish, belonging to 15 orders, 31 families and 58 genera, from the 73 commercial names previously recorded. We used both genetic similarity and phylogeny for molecular identification (Fig. 1). Many taxa did not match the commercial name assigned to them. The most diverse families were: Carangidae (12 species), Sciaenidae (10 species) and Ariidae (9 species) (Supplementary Table S1).

**Figure 2:**
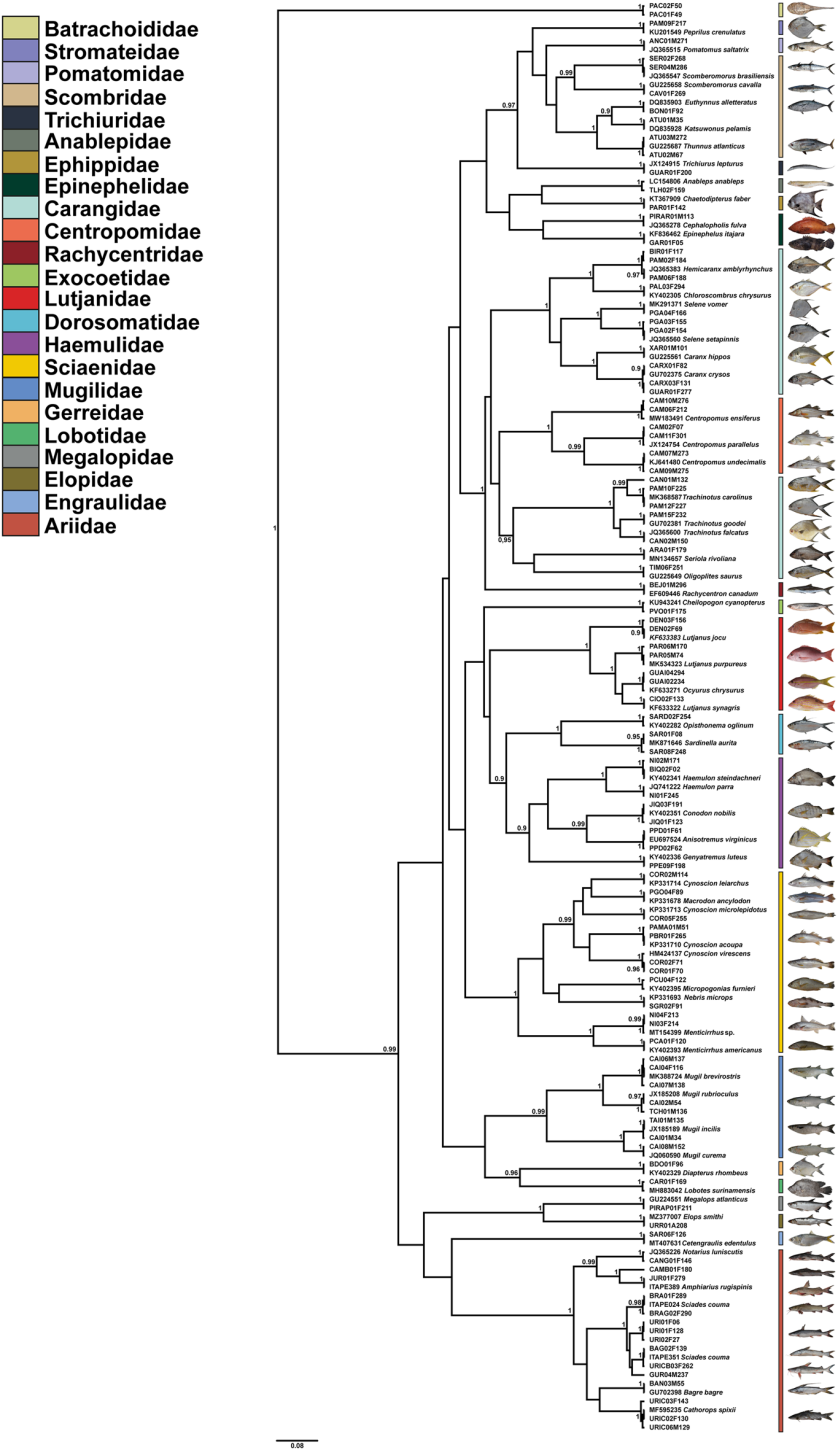
Bayesian inference tree for commercialized marine fish species at the Street Market of Bragança-PA. The numbers over the branches indicate statistical support. The coloring of taxa was organized by Family.

**Figure 3:**
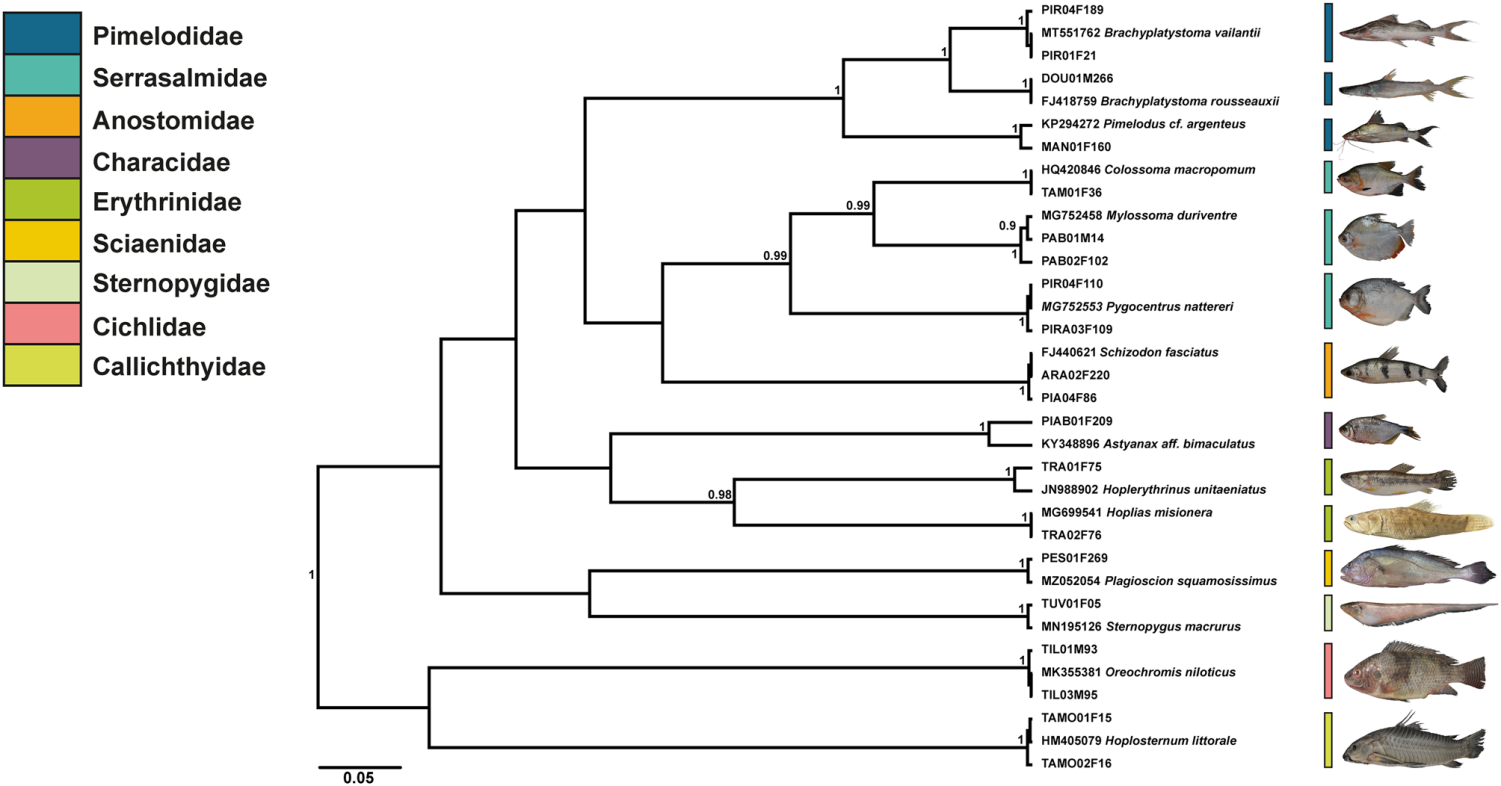
Bayesian inference tree for commercialized freshwater fish species at the Street Market of Bragança-PA. The numbers over the branches indicate statistical support. The coloring of taxa was organized by Family.

Most of the recorded species (68) were from marine and/or estuarine habitats (Fig. 2), while 14 were from freshwater (Fig. 3). We also report the first record of commercialization of *Haemulon atlanticus* (formerly known as *H. steindachneri*) (Family Haemulidae), *Menticirrhus cuiaranensis* (Sciaenidae) and *Hoplias misioneira* (Erythrinidae) species on the north coast of Brazil.

### Molecular identification

Using DNA Barcoding, we discriminated 82 species of fish from the Amazon region. We compared their genetic sequences with public databases and found 81 matches at the species level. The exceptions were *Aspistor quadriscutis* and *Batrachoides surinamensis*, which we identified by morphology and phylogenetic analysis. These two species had no reference sequences in public databases, so our sequences will serve as the first references for them (Table 1; Figs. 2, 3).

We also identified eight species by similarity only using the BOLD platform, but we did not include their sequences in our analyses because they came from a private source. They were *Trachinotus cayennensis*, *Cetengraulis edentulus*, *Hoplerythrinus unitaeniatus, Astyanax bimaculatus, Notarius grandicassis, Sciades parkeri, Sciades proops and Sciades herzbergii.*

Some samples had ambiguous identification results, such as the ones labeled as “cangatã”, which matched *Aspistor luniscutis* by similarity and *A. quadriscutis* by morphology. Other samples had high similarity with more than one species, such as “caica” 01 with *Mugil curema* and *Mugil rubrioculus*, “caica” 02 with *Mugil hospes* and *Mugil brevirostris*, “caica” 03 with *Mugil curema* and *Mugil trichodon*; the “gurijuba” sample with *Netuma sp*. and *S. parkeri*, “urubaiana” with *Elops smithi* and *Elops saurus* (Table 1). Another controversial case was found for the sequences of “bragalhão”, “bagre” and “uricica branca”, which the comparisons returned them ash *Sciades couma*, based on different sequences (ITAPE024 and ITAPE351). The clusters resulting from the NJ tree showed that “bragalhão” and the ITAPE024 sequence form a cluster and that the “bagre” and “uricica branca” form another clustering with the ITAPE351 sequence (Fig. 2). The two groups differ from each other with a divergence of 5.20%.

Mean genetic distances increased according to taxonomic level, with average of 0.13% within species, 11.55% within genera (between species) and 18.28% within families (between genera) respectively (Table 2). Intraspecific values ranged from 0.0% to 1.42%. The species showed barcode gaps, with a minimum distance between congeners of 4.16%, between *S. couma* and *S. props* (Table 2).

**Table 2:** Minimum, maximum, and average values of genetic divergence, using the K2P evolutionary model, among the sampled species, genera, and families.

	Taxa	Min dist (%)	Mean dist (%)	Max dist (%)
Within species	82	0.00	0.13	1.42
Within genus	58	4.16	11.55	21.21
Within family	31	7.08	18.28	27.47

### Trade names and endangered species

**Figure 4:**
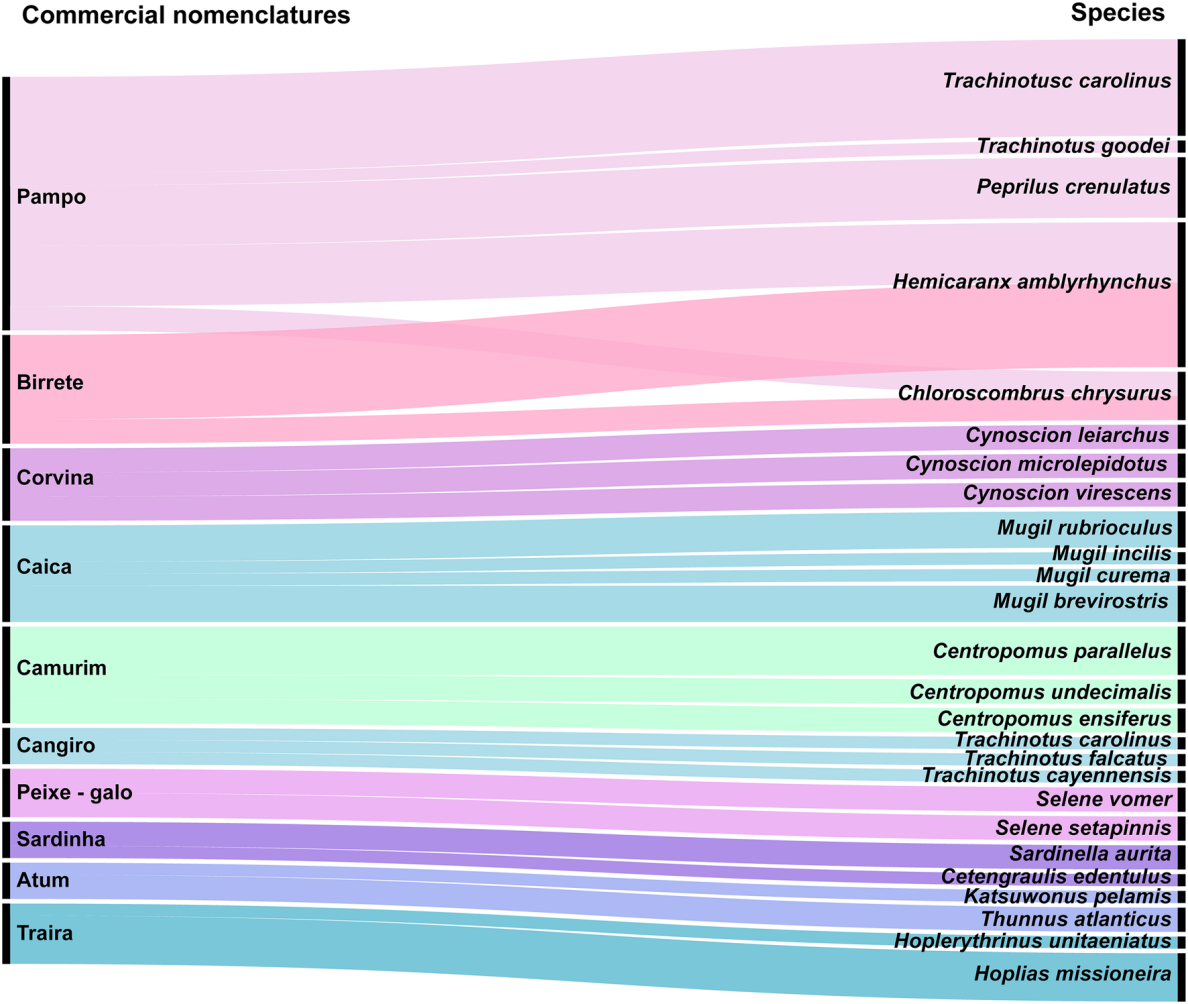
Alluvial diagram representing the 10 commercialization categories and the corresponding species. On the left side are the categories and on the right are the species.

73 trade names were identified in this study, corresponding to 82 species (Table 1). Among the designations, 10 were considered categories (Fig. 4), as they presented more than one species being sold by the same name. As an example, we have the “pampo” category, which had the highest number of species (n = 5), including *Chloroscombrus chrysurus, Hemicaranx amblyrhynchus, Trachinotus carolinus, Trachinotus goodei* and *Peprilus crenulatus* (Fig. 4).

Beside from this, 10 cases in which different trade names were used for the same species were also observed, such as *Cynoscion acoupa*, which has been sold as “pescada amarela”, “pescada branca” and “garoupa” (Table 1). On the other hand, the species *Haemulon parra, H. atlanticus* and *M. cuiaranensis* were found being sold without presenting a commercial name, therefore they were called “without commercial designation” (SDC).

The commercial designations, when compared with the correlation of common names and respective scientific names provided by Normative Instruction No. 53 of September 1, 2020 (MAPA), showed that there is compatibility for many of the identified species (47.6%), however, for other species, the names are different (23.4%), in addition to species found being commercialized, but which do not have a name on the MAPA list (28%) (Supplementary Table S1).

The study revealed five species with some level of threat, according to IUCN list (2023) and by Ordinance MMA nº 148^25^, including: the “mero” *Epinephelus itajara* (vulnerable—VU), “gurijuba” *S. parkeri* (vulnerable—VU), “pirapema” *Megalops atlanticus* (vulnerable—VU) and “ariacó” *Lutjanus synagris* (near threatened -NT)^26^. By MMA Ordinance No. 148, of June 7, 2022, *Epinephelus itajara* was considered (critically endangered-CR), *S. parkeri* (vulnerable—VU), *Megalops atlanticus* (vulnerable—VU) and “pargo” *Lutjanus purpureus* (vulnerable—VU)^25^.

## Discussion

This work represents the most comprehensive molecular analysis with Teleost fish traded in the coastal Amazon, with more than 200 individuals collected over four years of study. DNA barcoding tool was used to identify and validated the real commercialized diversity, masked due to the use of categories, and revealed an important trade of threatened species, in addition to species that its commercialization was first recorded in this study.

### DNA barcoding for ichthyodiversity identification

The DNA Barcoding tool was used to identify the fish diversity traded in Braganca. Comparisons were made with public database and phylogenetic trees, comprising 82 fish species, corresponding to the highest ichthyodiversity recorded to date, which was higher than those found by Braga et al.^9^ and Freire et al.^29^ and like the ones found by Martins et al.^12^, when considering only teleost fish. The identifications carried out in previous studies were based on vernacular nomenclatures and taxonomic keys, while in this study, we identified a large number of species commercialized in Bragança and in the North region through the DNA Barcoding approach, confirming the efficiency of the molecular tool to discriminate taxa, as observed in other studies with ichthyodiversity^24,27^, as well as to identification of processed products by the fishing industry^28^.

Within the identified species, the family Carangidae was the most representative with 12 species, contrary with previous studies that positioned the Sciaenidae family as the most representative^9,12,29^. Carangidae is constantly identified as one of the main families that composes the ichthyofauna of the Brazilian north coast^3,30^.

The entry of large number of Carangidae family species into local trade can be attributed to the emergence of a new market, during the closed season for “pargo” (*L. purpureus*) and other species of greater commercial value from the north coast of Brazil where the vessels are licensed for various fish species and many carangids, popularly known as 'black fish', are caught (personal communication). Another important fact is that this study is the first implementing the molecular approach on the diversity of teleost fish commercialized in Bragança, when compared to previous research that only used taxonomic keys^9,12,29^, leading the authors not to reach the identity of the evaluated species, thereby underestimating the group of Carangidae species sold.

In this study, the commercialization of *H. misioneira* in the North of Brazil was also observed for the first time. This species was described from the *Hoplias malabaricus* species complex, in the Uruguay, Paraguay and Paraná basins^31^ and according to Guimaraes et al.^24^, this species has a disjunct distribution, also occurring in the Amazon Basin. This is the second record outside its natural range, which shows that this species is probably distributed in other areas, since the *H. malabaricus* complex has a wide distribution^32^.

In addition, we recorded the trade of newly described species already being sold, and without having a commercial name, such as *M. cuiaranensis*, and *H. atlanticus*. This scenarios shows how the diversity of fish in the coastal Amazon is underestimated and misunderstood, since the capture and commercialization of this species was already happening even before we were aware of its presence. This is worrying, because while part of the biodiversity remains unknown, natural resources are being exploited at an increasingly accelerated pace^5^.

Probably, when it comes to fish, many taxa can be extinct even before being formally described due to the intense dynamics of capture and commercialization, with diverse and non-standard nomenclatures, associated with inefficient and/or non-existent inspection which strongly collaborate to reduce the biodiversity.

### Inconsistencies between morphological and molecular identifications

Ambiguities in the identifications were found for the “cangatã” fish, which molecular identification presented them as *A. luniscutis*, however, the species that is found in the Brazilian north region is *A. quadriscutis*. In addition, a study with a molecular and morphological approach showed that *Aspistor* species found on the Brazilian coast have morphological differences, but do not present significant genetic distances in mitochondrial genes such as cytochrome b (Cyt b) and subunits 8 and 6 of ATP synthase (ATPase 8/6)^33^. It may be that the same happens for the COI gene, causing the samples of *A. quadriscutis* to show great similarity with *A. luniscutis* and forming a clade in the phylogenetic tree (NJ).

For some species of the Ariidae family, the inconsistencies in the identifications probably occurred due to identification errors and consequent erroneous deposits in public database, as observed for the designations “bragalhão”, “bagre” and “uricica branca”, identified as *S. couma*, but which formed two distinct groups with a genetic distance of 5.20%, in the NJ tree, being “bragalhão” (clade 1), bagre” and “uricica branca” (clade 2), that was identified based on morphology as *S. couma* and *S. herzbergii* respectively.

Comparisons with public database showed ambiguity in identification. Cases like these were observed for the Mugilidae family, the species referring to “caica” 02, was 100% similar to *M. hospes* and *M. brevirostris* in the NCBI and BOLD. However, in the South Atlantic only *M. brevirostris* occurs^34^, therefore, the sequences deposited in Brazil as *M. hospes* are, considered *M. brevirostris*^35^. For the other members of the Mugilidae family, the identification was confirmed from the study conducted by Durand et al.^35^, where a new identification of sequences from the Mugilidae family deposited in Genbank was carried out, correcting erroneous deposits, therefore we identified “caica” 01 as *M. rubrioculus*, and “caica” 03 as *M. curema*.

Another case of incongruity was observed for the “urubaiana” fish, identified as *Elops smithi* and *Elops saurus* in public banks. However, Sousa et al. revealed the occurrence of only *E. smithi* on the Brazilian coast. Thus, the sequence assigned to *E. saurus* in Brazil is possibly a taxonomic error, which has already been reported for these two species in the literature^36,37^.

The samples considered to be *H. atlanticus* were identified in public database as *H. steindachneri,* before the description of *H. atlanticus* for the Western Atlantic, both belonging to the *H. steindachneri* complex^38^. Despite the study by Carvalho et al.^38^ used the genetic tool to confirm the existence of the two species, these sequences could not be used in this work, as they are not available in public databases.

Records of incompatibilities due to inaccurate or erroneous deposits in public database have been reported in the literature for both the BOLD system^27^ and the NCBI^34^. Faced with these shortcomings, researchers must be carefully when carrying out identifications by consulting specialized literature or specialists in each group or using reference database to resolve ambiguous cases, so that identification errors are mitigated and not perpetuated and reliability in the data deposited in public database is maintained.

Despite some ambiguities in species identifications, the DNA Barcoding tool was efficient in discriminating most of the taxa found in this study, with expressive Barcode gap. We recovered as the greatest intraspecific distance, (1.42%), found for the species *Mylossoma duriventre*, and the smallest interspecific distance (4.16%) between the species *Sciades couma* and *Sciades proops*.

### Commercial name and hidden diversity

82 species were found out of 73 trade names sampled, showing that there is no correspondence between the number of trade names and the number of traded species, since in some cases the designations act as a category and in others the same species receives different trade names. Marketing by category, as in Bragança and as it happens in most places, ends up with underestimating the fish diversity offered, mainly due to the difficulty of differentiating the taxa of some families with similar morphology, as observed for Centropomidae and its congeners sold as “camurim”, the *C. undecimalis*, *C. parallelus* and *C. ensiferus*; and Mugilidae sold as “caica”, *M. rubrioculus*, *M. brevirostris, M. curema,* and *M. incilis*.

An interesting case is the category “pampo” used for five species, including those from different genera (*C. chrysurus, H. amblyrhynchus*, *T. carolinus, T. goodei* and *P. crenulatus*) confirming that commercial designations do not offer precision about the commercialized species. Some commercial names describe large groups, configuring themselves as a category, this is already described for nomenclatures such as “pargo”, “bagre”, “sardinha”, “pescada”, “garoupa”, and “arraia”^17,39^.

Marketing through generalist names can pose a threat to fish conservation, as several species can be sold through categories, including endangered species^14,16,17^, as the case of *E. itajara* sold under the designation/category “garoupa”. It is important to note that the commercialization of *E. itajara* in Brazil has been prohibited since 2002^40^, therefore, the commercialization of this species is taking place illegally. The trade of endangered species is worrying, basically when it is facilitated by the non-standardization of the commercial nomenclature that masks this market.

One case of substitution occurred for fish sold under the name “pescada branca”, which according to normative instruction MAPA Nº 53 of 2020, should only be used for the species *Cynoscion leiarchus* and *Plagioscion squamosissimus*. However, all samples collected under this designation were identified as *Cynoscion acoupa*, a species normally sold as “pescada amarela” and which is of great commercial importance^7,12^. This replacement probably occurred accidentally, since the individuals collected were juveniles and many species of the Sciaenidae family are morphologically similar in the early stages and have a sympatric distribution, which can lead to difficulties in the correct identification of taxa, as already reported in other works^18^.

For continental species, we found different taxa being marketed through the designation “traíra”, including different genera, they are: “traíra” 01, identified as *Hoplerythrinus unitaeniatus* and “traíra” 02 *H. misioneira*.

Although the MAPA normative tries to establish and standardize the relationship between the common names and respective scientific names for the main commercialized species, it still has redundancies, as it provides several common names for a single species and in some cases displays nomenclatures for fish down to the genus level, such as “canguiro” and “pampo” for *Trachinotus sp*., opening space for permanence of categories. When comparing the Feira Livre designations with Normative Instruction No. 53 of September 1, 2020, we noticed that a range of taxa does not have a similar name in the normative instruction, as well as several species are not present in the list. This reveals that we have a document that needs to be revised to establish in a coherent and specific way the trade name and corresponding species in Brazil. The alternative to reduce the gaps left by categorization is the creation of lists by region, since the nomenclatures vary a lot, even in nearby places.

### The importance of knowing the diversity of fish in the trade

Trade in Bragança is predominantly carried out with marine species, but some freshwater species are also sold, including fish from fish farming such as “tilápia” *Oreochromis niloticus* and “Tambaqui” *Colossoma macropomum*.

We observed the trade of species that had not been previously registered in the Bragantina region^9,12,29^, including *Katsuwonus pelamis* (“atum”), *C. ensiferus* (“camurim”), *T. cayennensis* (“pampo”), *T. goodei* (“pampo”*), Selene setapinnis* (“peixe galo”), *H. parra* (SDC) and *M. cuiaranensis* (SDC). For freshwater fish we found *Schizodon fasciatus* (“aracu/piau”), *H. missioneira* (“traira”), *M. duriventre* (“pacu/paboca”) and *Pygocentrus nattereri* (“piranha”).

The results reveal that trade in Bragança is quite dynamic, with changes in the composition of species offered over the year (Table 3). Certainly, there are species that were not sampled, as the landings and commercialization of fish in Bragança occur daily^6^, however, this study presents the most complete data regarding the diversity of teleost fish commercialized in the Bragança region (Table 3).

**Table 3:** List of fish families and species sold at the Bragança Free Market over the years.

Family	Species of the present study	Location	2016/2017	2018/2019	Freire et al.^29^	Martins et al.^12^
Anablepidae	*Anableps anableps* (Linnaeus, 1758)	F	x		x	x
Anostomidae	*Schizodon fasciatus* Spix e Agassiz, 1829	F	x	x		x
Ariidae	*Amphiarius rugispinis* (Valenciennes, 1840)	F		x	x	x
	*Bagre bagre* (Linnaeus, 1766)	M/F	x	x	x	x
	*Cathorops spixii* (Agassiz, 1829)	F	x		x	x
	*Notarius grandicassis* (Valenciennes, 1840)	F		x	x	x
	*Aspistor quadriscutis* (Valenciennes, 1840	F	x	x	x	
	*Sciades couma (*Valenciennes, 1840)	F		x	x	x
	*Sciades parkeri* (Traill, 1832)	M/F	x	x	x	x
	*Sciades proops* (Valenciennes, 1840)	M/F	x	x	x	x
	*Sciades herzbergii* (Bloch, 1794)	M/F	x	x	x	
Batrachoididae	*Batrachoides surinamensis* (Bloch e Schneider, 1801)	F	x		x	x
Callichtyinidae	*Hoplosternum littorale* (Hancock, 1828)	F	x			x
Carangidae	*Caranx crysus* (Mitchill, 1815)	F	x	x		x
	*Caranx hippos* (Linnaeus, 1766)	M	x	x		x
	*Chloroscombrus chrysurus* (Linnaeus, 1766)	F		x		
	*Hemicaranx amblyrhynchus* (Cuvier, 1833)	F	x	x		x
	*Oligoplites saurus* (Bloch e Schneider, 1801)	F	x	x		
	*Selene setapinnis* (Mitchill, 1815)	F	x			
	*Selene vomer* (Linnaeus, 1758)	F	x			
	*Seriola rivoliana* Valenciennes, 1833	F		x		
	*Trachinotus carolinus* (Linnaeus, 1766)	F		x		
Carangidae	*Trachinotus cayennensis* Cuvier, 1832	M	x		x	
	*Trachinotus falcatus* (Linnaeus, 1758)	M	x			
	*Trachinotus goodei* Jordan and Evermann, 1896	F		x		
Centropomidae	*Centropomus parallelus* Poey, 1860	M/F	x	x		x
	*Centropomus ensiferus* Poey, 1860	M		x		
	*Centropomus udecimalis* Bloch, 1792	M		x		
Characidae	*Astyanax bimaculatus*	F		x		
Cichlidae	*Oreochromis niloticus* (Linnaeus, 1758)	M/F	x			
Dorosamatidae	*Opisthonema oglinum* (Lesueur, 1818)	F		x	x	
	*Sardinella aurita* Valenciennes, 1847	F	x	x		
Engraulidae	*Cetengraulis edentulus* (Cuvier, 1829)	F	x			
Elopidae	*Elops smithi* McBride, Rocha, Ruiz-Carus & Bowen, 2010	F		x		
Epinephelidae	*Cephalopholis fulva (*Linnaeus*, 1758)*	M	x			x
	*Epinephelus itajara (*Lichtenstein*, 1822)*	M/F	x	x		
Epheppidae	*Chaetodipterus faber* (Broussonet, 1782)	F	x	x	x	x
Erythrinidae	*Hoplias missioneira* Rosso, Mabragaña, González-Castro, Delpiani, Avigliano, Schenone e Díaz de Astarloa, 2016	F	x	x		
	*Hoplerythrinus unitaeniatus* (Spix e Agassiz, 1829)	F	x			x
Exocoetidae	*Cheilopogon cyanopterus (*Valenciennes*, 1847)*	F	x			
Gerreidae	*Diapterus rhombeus* (Cuvier, 1829)	M/F	x			
Haemulidae	*Anisotremus virginicus* (Linnaeus, 1758)	F	x			x
	*Conodon nobilis* (Linnaeus, 1758)	F	x	x		x
	*Genyatremus luteus* (Bloch, 1790)	M/F	x	x	x	x
	*Haemulon parra* (Desmarest, 1823)	F		x		
	*Haemulon atlanticus* Carvalho, Marceniuk, Oliveira & Wosiacki, 2020	F	x			
Lobotidae	*Lobotes surinamensis *(Bloch, 1790)	F	x	x		x
Lutjanidae	*Lutjanus jocu* (Bloch e Schneider, 1801)	M/F	x	x	x	x
	*Lutjanus purpureus* (Poey, 1866)	M	x		x	x
	*Lutjanus synagris* (Linnaeus, 1758)	F	x	x		x
	*Ocyurus chrysurus* (Bloch, 1791)	M	x			
Megalopidae	*Megalops atlanticus* Valenciennes, 1847	M		x	x	x
Mugilidae	*Mugil curema* Valenciennes 1836	M	x			
	*Mugil incilis* Hancock, 1830	M	x			x
	*Mugil brevirostris* (Ribeiro, 1915)	M/F	x			
	*Mugil rubrioculus* Harrison, Nirchio, Oliveira, Ron & Gaviria, 2007	M	x			
Pimelodidae	*Brachyplatystoma rousseauxii (*Castelnau*,* 1855*)*	M		x		
	*Brachyplatystoma vaillantii* (Valenciennes, 1840)	F	x	x	x	x
	*Pimelodus argenteus*	F	x	x	x	x
Pomatomidae	*Pomatomus saltatrix* (Linnaeus, 1766)	M		x		
Rachycentridae	*Rachycentron canadum* (Linnaeus, 1766)	M		x	x	x
Sciaenidae	*Cynoscion acoupa* (Lacepède, 1801)	M/F	x	x	x	x
	*Cynoscion leiarchus* (Cuvier, 1830)	M/F	x	x		
	*Cynoscion microlepidotu*s (Cuvier, 1830)	M/F	x	x	x	
	*Cynoscion virescens (Cuvier, 1830)*	M/F	x		x	x
	*Macrodon ancylodon* (Bloch e Schneider, 1801)	M/F	x	x	x	x
	*Menticirrhus americanus* (Linnaeus, 1758)	F	x		x	x
	*Menticirrhus cuiranaensis* (Marceniuk, Caires, Rotundo, Cerqueira, Siccha-Ramirez, Wosiacki e Oliveira, 2020*)*	M		x		
	*Micropogonias furnieri* (Desmarest, 1823)	M/F	x			x
	*Nebris microps* Cuvier, 1830	F	x		x	x
	*Plagioscion squamosissimus* (Heckel, 1840)	F		x		x
Scombridae	*Euthynnus alletteratus* (Rafinesque, 1810)	F	x	x		x
	*Katsuwonus pelamis (*Linnaeus, 1758*)*	M	x			
	*Thunnus atlanticus (*Lesson, 1831*)*	M		x		
	*Scomberomorus brasiliensis* Collette, Russo & Zavala-Camin, 1978	M/F	x	x	x	x
	*Scomberomorus cavalla* (Cuvier, 1829)	F		x		x
Serrasalmidae	*Colossoma macropomum* (Cuvier, 1816)	F	x			x
	*Mylossoma duriventre* (Cuvier, 1818)	F	x	x		x
	*Pygocentrus nattereri* Kner, 1858	F	x			
Sternopygidae	*Sternopygus macrurus* (Bloch & Schneider, 1801)	F	x	x		
Stromateidae	*Peprilus crenulatus* Cuvier, 1829	F	x	x		
Trichiuridae	*Trichiurus lepturus* Linnaeus, 1758	F		x	x	

With species collection location, “Mercado” (M) and/or “Feirinha” (F), biennium of sample collection 2016/2017 and/or 2018/2019 and comparison of the species in the present study with previous work carried out in the same locations: Freire et al.^29^ and Martins et al.^12^.

Our results shows the commercialization of species that were hidden by popular nomenclature and imprecise taxonomic identification. These data raise an alert about the capture and sale of species that already have low stocks, allowing the competent authorities to manage and supervise this market.

The measures for conservation and fisheries management be effective, it is first necessary to know the really diversity. We present here a list (Supplementary Table S1) with the correspondence between the commercial and biological designation for the species commercialized in Bragança, Amazonian coastal region, the first obtained through molecular identification and which will be an important tool for ordering the commercialization of fish in the region, considering all fish collected and molecular identifications carried out.

### Final considerations

In the present research, the DNA Barcoding tool proved to be extremely efficient for the discrimination and correct identification of the species sampled in Bragança. The results showed cases of replacement, trade of endangered species and unrecorded species diversity. Our results confirm that common and commercial names are inaccurate which underestimate ichthyodiversity and may favour replacements and trade of endangered species. Although we have a regulation to establish the relationship of commercial and specific names, it is incomplete, inefficient and needs a reformulation, which considers the diversity of names and the different Brazilian regions, to propose a standard name for each species. We therefore present a list of correspondence between trade name and referent species, considering the trade of Teleosts in the coastal portion of the Brazilian Amazon (Supplementary Table S1).

## Methods

### Ethics statement

All individuals were obtained from points of sale, they were already dead. There was no need to apply the guidelines of the Institution's Ethics Committee. In the same way, it was not necessary to obtain a collection license, since individuals were purchased during the commercialization process, or donated by traders.

### Sampling

The Bragança Free Market (“Feira Livre”) is the main place for selling fish, and is divided into two distinct environments, Market (“Mercado”) and Fair (“Feirinha”)^12^. The “Mercado” is supplied mainly by industrial fishing, aimed at target species of great commercial value, while the “Feirinha” is supplied mainly by artisanal fishing and that capture fish with less selectivity and greater diversity^9,12,19^. In this way, sample collection took place in both environments.

The collections were carried out monthly from April 2016 to February 2019, adding up to a total of 35 months of sampling. Fish samples were obtained through the purchase of whole individuals or donations from merchants. After tipping over, a sample of biological tissue was taken from each specimen. Three different tissues were used, depending on availability, tongue, fin and/or whole fish muscle. In this work, we adopted the term “commercial designation” to refer to the names observed during commercialization. For all collected commercial designations, whole individuals were fixed/preserved and incorporated, as exemplary testimonies, in the Ichthyological Collection of the Laboratory of Applied Genetics, of the Instituto of Coastal Studies, UFPA, Bragança. In addition, the photographic record has also been included for most commercial designations.

The whole acquired fish Specimens were identified to the taxonomic level possible, using morphological characters, through specialized literature^4,10,41^.

The biological tissue samples were stored in Eppendorf-type microtubes (2.0 mL), with 70% commercial alcohol and in a freezer at -20º C, for molecular analysis.

### Laboratory procedures

Genomic DNA was obtained using the commercial Wizard Genomic Kit (PROMEGA), following the manufacturer's instructions. After isolation, the samples were mixed with blue juice buffer solution and GelRed dye (2μL of the mixture and 2μL of DNA) and subjected to horizontal underwater electrophoresis in agarose gel (1%) for 30 min/60 V. After the electrophoretic run, the samples were visualized under ultraviolet light to verify the quality of the extracted DNA.

The COI gene target fragment was amplified by Polymerase Chain Reaction (PCR), using the FishF1 and FishR1 and FishF2 and FishR2 primers described by^42^. The reaction consisted of a final volume of 15μL, and the amplification conditions were those used by^42^, with modifications in the hybridization temperatures to 53º C and 54º C.

After PCR, the positive samples were purified with Polyethylene Glycol (PEG) according to the protocol by^43^ and submitted to the sequencing reaction, using the dideoxyterminal method^44^, with reagents from Big Dye Kit (ABI PrismTMDye Terminator Cycle Sequencing Reading Reaction—Thermo Fisher). The precipitated products were subjected to capillary electrophoresis in an ABI 3500XL automatic sequencer (Thermo Fisher).

### Sequences database and genetic analysis

The generated sequences were inspected manually, edited in the BioEdit v. 7.1.3.0^45^ and automatically aligned using the CLUSTAL W application^46^. The DNAsp v 6 program^47^ was used to generate a list of haplotypes, to assist in the sample identification process.

For the specimen identification process, each haplotype was initially compared to sequences available in public banks, GenBank Platforms, more specifically in the Basic Local Alignment Search Tool (BLAST), in the “nucleotide blast” field^48^ and BOLD (Barcoding of Life Database)^49^. The maximum divergence level adopted for individuals of the same species was 2%^22^.

Species reference sequences, available at NCBI and BOLD Systems, were added to the haplotype database generated in the work for the construction of the Neighbor Clustering tree, using the Kimura-2-parameters evolutionary model^50^, in MEGA 11^51^ program with the significance of the clusters estimated by Bootstrap analysis, 1000 pseudoreplicas^52^.

The maximum and average Barcode gap distances of the sequences were evaluated on the BOLD Systems platform (http://www.boldsystems.org/index.php/MAS_Management_DataConsole?codes=CA), through the Barcode Gap Analysis tool and the possible presence of stop codon were also checked in the same plataform. To complement the genetic distance data, we also used the MEGA 11 Program^50^, based on the K2P evolutionary model^49^.

The choice of the best evolutionary model for Bayesian inference trees was obtained on the CIPRES Science Gateway v3.3 platform^53^, using jModelTest2 in XSEDE^54^, the analysis recommended the HKY +|I + G evolutionary model for the marine and freshwater species bank, based on the Bayesian Information Criterion (BIC).

The construction of the Bayesian inference tree (BI) was performed using BEAST v. 1.8.4^55,56^. In the construction of the trees, a strict clock and the Yule speciation process were used. The posterior probability was estimated with three million generations and 10% burn-in. The log files were checked in Tracer v1.7.2 tool^57^ to assess chain convergence and proper burn-in length. The convergence chains considered adequate showed a value greater than > 200 ESS (effective sample size). Trees generated in BEAST were summarized in TreeAnnotator, v1.10.4, to obtain the best tree. FigTree, v1.4.4^58^ was used to visualize the resulting tree.

### Comparison of trade designations and threat status

Based on species identification, we compared the list of commercial names and corresponding species with the list of common names and respective scientific names provided by Normative Instruction No. 53 of September 1, 2020 (MAPA). We verified the threat status of each species in the red list of the International Union for the Conservation of Nature (IUCN) and in the Official List of Threatened Species of Brazil, Ordinance of the Ministry of the Environment no. 148, of June 07, 2022 (MMA no. 148)^25^.

The graph used to illustrate the categories obtained in this study was created in RAWGraphs (https://rawgraphs.io/).

### Supplementary Information

Supplementary Table 1.

## Data Availability

The data sets generated during and analyzed during the present study are available in the repository of the National Center for Biotechnology Information, with the codes OR459502-OR459617 and OR515260-OR515262.
