# Supplementary material for: DNA barcode reveals occurrence of threatened species and hidden diversity on Teleost fish trade in the Coastal Amazon
Source: Sci Rep. 2023 Nov 13;13:19749. doi: 10.1038/s41598-023-47063-2 (PMC10643451; doi:10.1038/s41598-023-47063-2)
Supplement: Supplementary file 1 — Supplementary Table 1. [file 41598_2023_47063_MOESM1_ESM.docx]

**Supplementary Table 1**. List of species sold at Feira Livre de Bragança, with commercial designation and names found in normative instruction No. 53 of September 1, 2020 (MAPA)

| FAMILY | SPECIES | COMERCIAL DESIGNATION | | IN. Nº 53 de 2020 (MAPA) |
| --- | --- | --- | --- | --- |
| Anablepidae | *Anableps anableps* (Linnaeus 1758) | "tralhoto" | Does not own | |
| Anostomidae | *Schizodon fasciatus* Spix e Agassiz 1829 | "piau"/ "aracu" | "aracu", "araçu" | |
| Ariidae | *Amphiarius rugispinis* (Valenciennes, 1840) | "jurupiranga" | "bagre", "bagre-jurupiranga", "jurupiranga" | |
|  | *Bagre bagre* (Linnaeus, 1766) | "bandeirado" | "bagre", "bagre-de-penacho", "bagre-bandeirado", "bandeirado" | |
|  | *Cathorops spixii* (Agassiz, 1829) | "uricica amarela" | "bagre", "bagre-amarelo", "bagre-uricica", "uricica" | |
|  | *Notarius grandicassis* (Valenciennes, 1840) | "cambéua" | "bagre", "bagre-branco", "bagre-cambeba", "cambeba", "bagre-cambéu", "cambéu", "bagre-cambéua", "cambéua", "bagre-bam"- "beua", "cambeua" | |
|  | *Aspistor quadriscutis* (Valenciennes 1840 | "cangatã" | "bagre", "bagre-cangatá", "cangatá", "bagre-cangatã", "cangatã" | |
|  | *Sciades couma* (Valenciennes, 1840) | "bragalhão" | "bagre", "bagre-catinga", "bagre-cuma" | |
|  | *Sciades parkeri (*Traill, 1832) | "gurijuba" | "bagre", "bagre-gurijuba", "gurijuba" | |
|  | *Sciades proops* (Valenciennes, 1840) | "uritinga" | "bagre", "bagre-uritinga", "uritinga", "bagre-urutinga", "urutinga" | |
|  | *Sciades herzbergii* (Bloch, 1794) | "bagre/uricica branca" | Does not own | |
| Batrachoididae | *Batrachoides surinamensis* (Bloch e Schneider*,* 1801) | "pacamum" | Does not own | |
| Callichtyinidae | *Hoplosternum littorale* (Hancock, 1828) | "tamuatá" | Does not own | |
| Carangidae | *Caranx crysus* (Mitchill, 1815) | "caraximbó/ guarajuba" | "xaréu", "xarelete", "xerelete", "carapau", "garajuba" | |
|  | *Caranx hippos* (Linnaeus, 1766) | "xaréu" | "xaréu", "xarelete", "xerelete", "carapau", "xaréu-cabeçudo", "xere- lete"-"cabeçudo", "xarelete-cabeçudo" | |
|  | *Chloroscombrus chrysurus* (Linnaeus, 1766) | "palombeta/ birrete/ pampo" | "palombeta" | |
|  | *Hemicaranx amblyrhynchus* (Cuvier, 1833) | "birrete/pampo" | Does not own | |
|  | *Oligoplites saurus* (Bloch e Schneider*,* 1801) | "timbiro" | Does not own | |

| FAMILY | | SPECIES | COMERCIAL DESIGNATION | | IN. Nº 53 de 2020 (MAPA) |
| --- | --- | --- | --- | --- | --- |
| Carangidae | *Selene* *setapinnis* (Mitchill, 1815) | | | “peixe-galo” | Does not own |
|  | *Selene vomer* (Linnaeus, 1758) | | | “peixe-galo” | “peixe-galo-de-penacho” |
|  | *Seriola rivoliana* Valenciennes, 1833 | | | “arabaiana” | Does not own |
|  | *Trachinotus carolinus* (Linnaeus, 1766) | | | “canguiro/pampo” | Does not own |
|  | *Trachinotus cayennensis* Cuvier, 1832 | | | “pampo” | Does not own |
|  | *Trachinotus falcatus* (Linnaeus, 1758) | | | “canguiro” | Does not own |
|  | *Trachinotus goodei* Jordan e Evermann, 1896 | | | “pampo” | “pampo”, “canguira”, “pampo-listrado” |
| Centropomidae | *Centropomus parallelus* Poey, 1860 | | | “camurim” | “robalo”, “robalo-peva”, “camurim”, “camorim” |
|  | *Centropomus* *ensiferus* Poey, 1860 | | | “camurim” | Does not own |
|  | *Centropomus udecimalis* Bloch, 1792 | | | “camurim” | “robalo”, “robalo-flecha”, “camurim”, “camorim” |
| Characidae | *Astyanax bimaculatus* | | | “piaba” | “lambari” |
| Cichlidae | *Oreochromis niloticus* (Linnaeus, 1758) | | | “tilápia” | “tilápia”, “tilápia-do-nilo” |
| Dorosomatidae | *Opisthonema oglinum* (Lesueur, 1818) | | | “sarda” | “sardinha-laje” |
|  | *Sardinella aurita* Valenciennes, 1847 | | | “sardinha” | “sardinha” |
| Engraulidae | *Cetengraulis edentulus* (Cuvier, 1829) | | | “sardinha” | “manjuba-boca-torta”, “boca-torta” |
| Elopidae | *Elops smithi* McBride, Rocha, Ruiz-Carus & Bowen, 2010 | | | “urubaiana” | Does not own |
| Epinephelidae | *Cephalopholis fulva* (Linnaeus, 1758) | | | “pirarena” | “piraúna”, “catuá” |
|  | *Epinephelus itajara (*Lichtenstein*, 1822)* | | | “garoupa/mero” | “mero” |
| Epheppidae | *Chaetodipterus faber* (Broussonet, 1782) | | | “parú” | “paru”, “paru-branco”, “enchada” |
| Erythrinidae | *Hoplias missioneira* Rosso, Mabragaña, González-Castro, Delpiani, Avigliano, Schenone e Díaz de Astarloa, 2016 | | | “traíra” | Does not own |
|  | *Hoplerythrinus unitaeniatus* (Spix e Agassiz, 1829) | | | “traíra” | “Jeju” |

| FAMILY | SPECIES | COMERCIAL DESIGNATION | | | IN. Nº 53 de 2020 (MAPA) | |
| --- | --- | --- | --- | --- | --- | --- |
| Exocoetidae | *Cheilopogon cyanopterus (*Valenciennes*, 1847)* | | “peixe-voador” | “peixe-voador”, “peixe-voador-holandê” | |  |
| Gerreidae | *Diapterus rhombeus* (Cuvier, 1829) | | “bico doce” | “carapeba” | |  |
| Haemulidae | *Anisotremus virginicus* (Linnaeus, 1758) | | “peixe pedra doido” | “salema”, “mercador” | |  |
|  | *Conodon nobilis* (Linnaeus, 1758) | | “jiquirí” | “roncador”, “coró”, “coró-roncador” | |  |
|  | *Genyatremus luteus* (Bloch, 1790) | | “peixe-pedra” | “coró”, “peixe-pedra”, “golosa” | |  |
|  | *Haemulon parra* (Desmarest, 1823) | | “não identificado” | “cambuba”, “xira”, “biquara” | |  |
|  | *Haemulon atlanticus* Carvalho, Marceniuk, Oliveira & Wosiacki, 2020 | | “biquara/ não identificado” | “cocoroca-boca-larga” | |  |
| Lobotidae | *Lobotes surinamensis*(Bloch, 1790) | | “carauaçu” | “prejereba”, “xancarrona”, “chancarana”, “curuaçu” | |  |
| Lutjanidae | *Lutjanus jocu* (Bloch e Schneider, 1801) | | “dentão” | “vermelho”, “dentão” | |  |
|  | *Lutjanus purpureus* (Poey, 1866) | | “pargo” | “vermelho”, “pargo”, “pargo-vermelho” | |  |
|  | *Lutjanus synagris* (Linnaeus, 1758) | | “cioba” | “vermelho”, “ariacó” | |  |
|  | *Ocyurus chrysurus* (Bloch, 1791) | | “guaiúba” | “vermelho”, “guaiúba”, “cioba” | |  |
| Megalopidae | *Megalops atlanticus* Valenciennes, 1847 | | “pirapema” | “tarpon”, “tarpão”, “camurupim”, “pema”, “pirapema” | |  |
| Mugilidae | *Mugil curema* Valenciennes 1836 | | “caíca” | “parati”, “saúna”, “pratiqueira”, “parati-cara-amarela” | |  |
|  | *Mugil incilis* Hancock, 1830 | | “caíca/tainha” | Does not own | |  |
|  | *Mugil brevirostris* (Ribeiro, 1915) | | “caíca” | Does not own | |  |
|  | *Mugil rubrioculus* Harrison, Nirchio, Oliveira, Ron & Gaviria, 2007 | | “caica/ tainha chata” | Does not own | |  |
| Pimelodidae | *Brachyplatystoma rousseauxii (*Castelnau*,* 1855*)* | | “dourada” | Does not own | |  |
|  | *Brachyplatystoma vaillantii* (Valenciennes, 1840) | | “piramutaba” | “piramutaba” | |  |
|  | *Pimelodus argenteus* | | “mandii” | “pintadinho”, “mandi” | |  |
| Pomatomidae | *Pomatomus saltatrix* (Linnaeus, 1766) | | “anchova” | “enchova”, “anchova” | |  |

| FAMILY | | SPECIES | | COMERCIAL DESIGNATION | | IN. Nº 53 de 2020 (MAPA) |  |
| --- | --- | --- | --- | --- | --- | --- | --- |
| Rachycentridae | *Rachycentron canadum* (Linnaeus, 1766) | | “beijupirá” | | Does not own | | |
| Sciaenidae | *Cynoscion acoupa* (Lacepède, 1801) | | “pescada amarela/branca” “garoupa” | | “pescada”, “pescada-amarela”, “pescada-jaguara” | | |
|  | *Cynoscion leiarchus* (Cuvier, 1830) | | “corvina” | | “pescada”, “pescada-branca” | | |
|  | *Cynoscion microlepidotu*s (Cuvier, 1830) | | “corvina” | | “pescada”, “pescada-dentão”, “pescada-bicuda” | | |
|  | *Cynoscion virescens (Cuvier, 1830)* | | “corvina” | | “pescada”, “pescada-cambucu”, “pescada-cambuçu”, “pescada-cambuci”, “pescada-corvina” | | |
|  | *Macrodon ancylodon* (Bloch e Schneider, 1801) | | “gó” | | “pescadinha”, “pescadinha-real”, “pescada-gó”, “pescada-foguete” | | |
|  | *Menticirrhus americanus* (Linnaeus, 1758) | | “pau de cachorro” | | Does not own | | |
|  | *Menticirrhus cuiranaensis* (Marceniuk, Caires, Rotundo, Cerqueira, Siccha-Ramirez, Wosiacki e Oliveira, 2020*)* | | “não identificado” | | Does not own | | |
|  | *Micropogonias furnieri* (Desmarest, 1823) | | “pescada cururuca” | | “corvina”, “curuca”, “cascote”, “cururuca” | | |
|  | *Nebris microps* Cuvier, 1830 | | “sete grudes” | | “pescada-banana” | | |
|  | *Plagioscion squamosissimus* (Heckel, 1840) | | “pescadinha” | | “pescada-branca”, “pescada-do-piauí” | | |
| Scombridae | *Euthynnus alletteratus* (Rafinesque, 1810) | | “bonito” | | “bonito”, “bonito-pintado” | | |
|  | *Katsuwonus pelamis (*Linnaeus, 1758*)* | | “atum” | | “bonito-listrado”, “bonito-barriga-listrada”, “gaiado” | | |
|  | *Thunnus atlanticus (*Lesson, 1831*)* | | “atum” | | “atum”, “albacorinha” | | |
|  | *Scomberomorus brasiliensis* Collette, Russo & Zavala-Camin, 1978 | | “serra” | | “cavala”, “serra”, “sororoca” | | |
|  | *Scomberomorus cavalla* (Cuvier, 1829) | | “cavala” | | “cavala”, “cavala-verdadeira” | | |
| Serrasalmidae | *Colossoma macropomum* (Cuvier, 1816) | | “tambaqui” | | “Tambaqui” | | |
|  | *Mylossoma duriventre* (Cuvier, 1818) | | “pacu/paboca” | | Does not own | | |
|  | *Pygocentrus nattereri* Kner, 1858 | | “piranha” | | Does not own | | |
| Sternopygidae | *Sternopygus macrurus* (Bloch & Schneider, 1801) | | “tuvi” | | Does not own | | |
| Stromateidae | *Peprilus crenulatus* Cuvier, 1829 | | “pampo” | | “gordinho”, “canguiro” | | |
| Trichiuridae | *Trichiurus lepturus* Linnaeus, 1758 | | “guaravilha” | | “peixe-espada” | | |
